# ftmsRanalysis: An R package for exploratory data analysis and interactive visualization of FT-MS data

**DOI:** 10.1371/journal.pcbi.1007654

**Published:** 2020-03-16

**Authors:** Lisa M. Bramer, Amanda M. White, Kelly G. Stratton, Allison M. Thompson, Daniel Claborne, Kirsten Hofmockel, Lee Ann McCue

**Affiliations:** 1 Computational Analytics Division, Pacific Northwest National Laboratory, Richland, Washington, United States of America; 2 Environmental Molecular Sciences Division, Pacific Northwest National Laboratory, Richland, Washington, United States of America; Hebrew University of Jerusalem, ISRAEL

## Abstract

The high-resolution and mass accuracy of Fourier transform mass spectrometry (FT-MS) has made it an increasingly popular technique for discerning the composition of soil, plant and aquatic samples containing complex mixtures of proteins, carbohydrates, lipids, lignins, hydrocarbons, phytochemicals and other compounds. Thus, there is a growing demand for informatics tools to analyze FT-MS data that will aid investigators seeking to understand the availability of carbon compounds to biotic and abiotic oxidation and to compare fundamental chemical properties of complex samples across groups. We present *ftmsRanalysis*, an R package which provides an extensive collection of data formatting and processing, filtering, visualization, and sample and group comparison functionalities. The package provides a suite of plotting methods and enables expedient, flexible and interactive visualization of complex datasets through functions which link to a powerful and interactive visualization user interface, Trelliscope. Example analysis using FT-MS data from a soil microbiology study demonstrates the core functionality of the package and highlights the capabilities for producing interactive visualizations.

## Author summary

High-resolution mass spectrometry instruments provide a mechanism for researchers to better understand the fundamental chemical composition of materials such as soil, plants, petroleum, and beverages. The large and complex data generated by analysis of these materials has led to a growing demand for software tools to aid researchers in processing, analyzing, and creating informative visualizations of these data. To move beyond existing software tools designed for specific purposes and visualizations of data from an individual sample, we present a software package, *ftmsRanalysis*, that provides researchers with a large collection of methods for streamlining the downstream processing high-resolution mass spectrometry data. *ftmsRanalysis* provides methods to compute useful chemical properties, filter data, define groups of samples, statistically compare sample groups, and make visualizations for many samples simultaneously. In this paper, we give an overview of *ftmsRanalysis’* general structure and capabilities. We then apply *ftmsRanalysis* to a soil microbiology dataset and present some of the results and visualizations generated by using the software package.

This is a *PLOS Computational Biology* Software paper.

## Introduction

Fourier transform mass spectrometry (FT-MS) analysis has become the preferred measurement platform for complex organic mixtures due to the high-resolution and mass accuracy that can be achieved [1–3]. The increasing use of FT-MS analysis resulted in over 240 publications last year in American Chemical Society journals alone, and FT-MS analyses have been used to examine a wide range of complex mixtures, including soils [4–7], plants [8], aquatic samples [9–11], petroleum [12] and various beverages [13–15].

FT-MS instruments produce spectral data, and depending on the sample type and instrument type, each spectrum contains thousands to tens of thousands of peaks observed across a range of masses. The increased demand for, and generation of, FT-MS data has led to increased research and development of proprietary and open source software to process the data, determine the unique mass value of peaks and assign molecular formulae [16, 17]. With high magnetic field instruments (e.g., 21 Tesla FT-MS), the data matrix for multiple samples of an experiment typically contains hundreds of thousands of identified peaks. The volume of the data generated by these instruments therefore presents a significant challenge for researchers in terms of data processing, filtering, exploratory analysis and visualization. To date, most researchers have utilized custom dataset-specific scripts and pipelines for the downstream processing and visualization of FT-MS data, and the availability of open-source, reproducible processing pipelines has been limited to specific visualizations (e.g. [18]). The first browser-based application was recently released [19] with a pipeline for processing FT-MS data.

In this manuscript, we introduce *ftmsRanalysis*, an R package which fills the existing gap in this field by providing users with a large collection of formal methods for streamlining the downstream processing of FT-MS data. The package is applicable to FT-MS data, Fourier transform ion cyclotron resonance (FTICR) data, and any mass spectrometry data for which minimal elemental information are available. The intended users of this R package are scientists who are analyzing data provided to them by mass spectrometry experts, after formula assignment has been performed. Novel aspects of the package include: standard and custom filtering methods, statistical comparisons and visualizations beyond single samples (e.g. comparisons of experimental groups), and the capability to deploy interactive visualizations for samples and groups into a graphical user interface for simultaneous visualization, with the capability to sort and filter plots according to calculated metrics. We demonstrate the use of the package on a soil microbiology dataset and present exemplar visualizations produced by *ftmsRanalysis* for these data.

### Design and implementation

The *ftmsRanalysis* open-source software package provides exploratory analysis and visualization tools for FT-MS data, using simple function calls that operate on defined data objects. Capabilities include the calculation of chemical properties based on the molecular formulae of observed compounds, filtering based on characteristics of the data, and the creation of various plots that can be used to further understand the dataset. Here, we provide brief descriptions of the capabilities of *ftmsRanalysis;* additional details are available in the package vignettes. Additionally, documentation of all functions organized by capability can be found online (https://emsl-computing.github.io/ftmsRanalysis/reference/).

### Data format

The primary data class in the *ftmsRanalysis* package is an S3 data object: peakIcrData. S3 data objects in R have predefined structures with properties that allow a developer to write object-specific methods; for instance, the plot() command will display different types of plots when called on different types of S3 objects. Therefore, functions in the *ftmsRanalysis* package are written to operate on peakIcrData.

The peakIcrData object consists of the following three data tables, each of which corresponds to a data.frame in R and can be imported using R’s base functionality. Table 1(A)–1(C) gives example structures of the three data.frames. (1) e_data is a *p* by (*n* + 1) data.frame containing the peak intensity data from an FT-MS instrument, where *p* is the number of observed peaks and *n* is the number of samples. Each row of e_data corresponds to the molecular mass for a peak and one column specifies its unique identifier, typically its mass value. (2) f_data is a data.frame with *n* rows, each corresponding to a sample. One column specifies the unique sample identifier used as the column names of e_data, and the remaining columns contain qualitative and/or quantitative traits of each sample. (3) e_meta is a data.frame with *p* rows corresponding to the rows of e_data. Thus, one column specifies the unique identifiers in e_data, and the remaining columns specify the molecular formula assignments, either as the molecular formula in a single column or individual columns containing counts for each of the elements. *ftmsRanalysis* assumes that elemental counts are available for Carbon (C) and Hydrogen (H) and allows for counts of Oxygen (O), Nitrogen (N), Sulfur (S), and Phosphorus (P). If isotopic peaks (e.g. ^13^C) were detected for a given mass and formula assignment, e_meta can include a column that indicates presence/absence of the isotope (“1”, “yes”, “TRUE”, etc.). In this case, the definition of the peakIcrData object will include specification of the presence of isotopic peak information in e_meta as well as the indicator used.

**Table 1:** Example format of required input data.frames for ftmsRanalysis package showing the first few rows and columns of each.

*(a) e_data*				*(b) e_meta*		*(c) f_data*		
mass	sample 1	sample 2	…	mass	molecular formula	sample	Location	Vegetation
**221.06665**	1152257.5	1812663.0	…	**221.06665**	C8H14O7	**sample 1**	Michigan	Corn
**222.00432**	2158339.3	0	…	**222.00432**	C9H5O6N1	**sample 2**	Wisconsin	Switchgrass

### Preprocess & summarize

The *ftmsRanalysis* package operates on the peakIcrData object for preprocessing and summarization of the data. If the peakIcrData object was defined using elemental counts, the assign_mf() function is automatically implemented to augment e_meta with the molecular formulae based on those counts; for example, if for a given mass the individual elemental columns indicate six C atoms, twelve H atoms, and six O atoms, then the formula C_6_H_12_O_6_ will be added to e _meta. The data can be transformed from the raw peak intensities to presence/absence data, or log transformed (in this case values of zero are replaced with NA). If the peakIcrData object was defined using the molecular formulae, the parse_mf() function is automatically implemented to add elemental count columns to e_meta. The group_designation() function can be used to associate samples to particular groups (e.g., experimental treatment groups), using the f_data data.frame. The defined groups are utilized by plot() and summary() methods as well as downstream visualization functions.

At any point during data processing, the summary() and plot() functions can operate on the peakIcrData object. The summary() of a peakIcrData object returns the number of samples, the number of identified masses and the number of assigned formulae in the dataset, as well as the percent of values that are missing. If group_designation() has been run on the peakIcrData object, summary() also returns the number of samples per group. Depending on the scale of the data (intensity, presence/absence, log transformed), plot() displays different types of graphs color-coded by group, if applicable. For instance, when data are represented by peak intensities, plot() displays boxplots of the peak intensities in each sample, and when data are on the presence/absence scale, plot() displays bar graphs for the number of observed masses in each sample. If groups have been associated with the samples in the dataset, the plot() function will order the samples so that samples belonging to the same group are organized together on the plot.

### Calculate

When exploring and analyzing FT-MS data, certain calculated values related to chemical properties may be of interest, thus *ftmsRanalysis* calculates several frequently-used values via the compound_calcs() function. These include several elemental ratios (e.g. O:C, H:C, etc.), Kendrick mass and defect, nominal oxidation state of carbon (NOSC), Aromaticity Index (AI) and modified AI, the double bond equivalent (DBE) and DBE minus oxygen (DBE—O) [20, 21] and Gibbs free energy of the Carbon oxidation half reaction under standard conditions [22]. The compound_calcs() function takes as input the peakIcrData object and a vector of function names, as denoted in Table 2. The function returns a peakIcrData object where the e_meta data.frame has been augmented with the specified information (e.g., if calc_kendrick is called then columns containing the Kendrick mass and defect are added to the e_meta data.frame).

**Table 2:** Chemical property calculations available within ftmsRanalysis.

Name(s)	Function Name	Optional Arguments
Elemental ratios (O:C, H:C, N:C, P:C, N:P)	calc_element_ratios	–
Kendrick Mass and Defect	calc_kendrick	Compound Base: CH_2_ (default), CO_2_, O_2_, CHO
Nominal oxidation state of carbon (NOSC)	calc_nosc	–
Cox Gibbs Free Energy	calc_gibbs	–
Aromaticity Index (AI) and modified AI	calc_aroma	–
Double bond equivalent (DBE) and DBE minus oxygen (DBE—O)	calc_dbe	Covalency of each element C: 4, H: 1, N: 3, P: 3 (default)

Classification of compounds based on their elemental composition is done using the assign_elemental_composition() function. This function assigns an elemental composition class to each mass with a molecular formula based on the combination of carbon, hydrogen, oxygen, nitrogen, sulfur, and phosphorus in the formula. The compound formulae can be associated with potential higher-order classes (e.g., protein, carbohydrate, lipid, etc.) using assign_class() with a user-specified choice of boundary set [4, 23, 24].

### Filter

The peakIcrData objects can be filtered on several criteria. Identified masses or formulae can be filtered using any combination of the following: (1) the mass_filter() function will retain mass values within a specified range; (2) the molecule_filter() function will retain masses observed in a minimum number of samples; (3) the formula_filter() function will retain only those masses with an assigned molecular formula; and (4) the emeta_filter() is a versatile filter that will retain masses belonging to a user-specified category(ies) or having values within a user-specified numeric range for a specified column in e_meta. For example, the emeta_filter() function could be used to retain peaks within a specific range of Kendrick mass defects. In addition, samples or groups can be filtered using the subset() function which retains specific samples or groups, if groups were defined using the group_designation() function.

To apply a filter, a filter object must first be created via one of the four filter functions described above. Calling plot() and summary() on a filter object along with specific bounds or values provides a visual or numeric summary, respectively, of the effect of applying the specified filter. For instance, after a mass filter is created, summary() can be called on the mass filter object with min_mass and max_mass specified to see how many peaks would be retained if the filter was applied with the specified minimum and maximum masses. The plot() of the same mass filter object will display a histogram of the number of peaks associated with each mass window, and is colored to show how many peaks would be retained with the specified parameters. Filters can be applied to the data via the applyFilt() function.

### Mapping to metabolic pathways

Peaks with formulae assigned can be mapped to compounds in MetaCyc [25], a metabolic pathway database, using the mapPeaksToCompounds() function and the R package MetaCycData, which is available on Github. Although fully confident compound identification requires additional information beyond that provided by FT-MS data, the MetaCyc mapping function allows users to explore potential compound identities present in their data. Matches in the MetaCyc database can then be further mapped to reactions and pathways/super-pathways via the mapCompoundsToReactions() and mapCompoundsToModules() functions. This mapping functionality can be used to summarize pathways and reactions of interest and suggest further experiments to confirm the presence of specific compounds in the samples.

### Visualize

All graphics in *ftmsRanalysis* are generated using plotly [26] for R, which creates interactive web graphics using R code. This enables users to hover over points in plots and see details about the values graphed, zoom in and out, hide or display points, as well as generate publication-quality graphics. Plots can be generated for single samples, for a group of samples or for a comparison of two groups of samples. To generate plots for one or two groups, the summarizeGroups() function must be used to first create a new peakIcrData object that represents the group(s) of interest, and the plotting functions are applied to the new group-level data object. The summarizeGroups() function calculates group-level summaries per peak, such as the number or proportion of samples in which each mass is observed. The result is a new peakIcrData object, whose e_data element contains one column per group, per summary function. A statistical comparison of two groups, determining which compounds were uniquely observed in each group, can also be run via the divideByGroup() and summarizeGroupComparisons() functions. A user must specify the proportion or number of samples in which a compound must be observed to be considered present in that group. The compounds uniquely observed in one group can then be determined by a user specified threshold for absence or by conducting a formal qualitative statistical test, the G-test [27], to determine if the observation pattern of a compound in samples is associated with group membership. Plotting functions can then produce graphs with points colored by groups for which they were uniquely or non-uniquely observed.

Common FT-MS plots, the van Krevelen diagrams and Kendrick plots, can be generated by the vanKrevelenPlot() and kendrickPlot() functions, respectively. The van Krevelen plot is an H:C ratio versus O:C ratio scatter plot and the Kendrick plot is a scatter plot of Kendrick mass defect versus Kendrick mass. Both van Krevelen and Kendrick plots can be colored by values from e_meta, such as NOSC or AI. In addition, density plots [densityPlot()] are available, which show the histogram and/or density curve for the specified chemical property values from e_meta. Finally, custom scatter plots can be made [scatterPlot()] based on two user-specified values from e_meta.

Additionally, *ftmsRanalysis* enables a user to leverage the R package Trelliscope [28], which provides a flexible and scalable way to divide datasets into meaningful segments and apply methods–plots, quantitative and qualitative summaries–to each piece. The result is a user interface where one can arrange plots in a grid and interactively sort, filter, and query plots based on metrics of interest. Data visualization using Trelliscope requires the user to define both a panel function and a cognostics function that are applied to each subset of data. A panel function takes a data subset and constructs a plot (or panel) from it. Panel functions may construct plots in any plotting package used in R; *ftmsRanalysis* uses plotly and provides a wrapper function [panelFunctionGenerator()] which can be used with the package’s plotting methods [e.g. vanKrevelenPlot()] to make panel functions for Trelliscope. A cognostics function calculates summary statistics on each subset of data. These statistics are then provided in the Trelliscope user interface for sorting and filtering. Example cognostics could include data quantiles, related metadata or even links to external web resources. The cognostics functions [vanKrevelenCognostics(), kendrickCognostics(), and densityCognostics()] in *ftmsRanalysis* are designed to provide default cognostics relevant to Van Krevelen plots, Kendrick plots and density plots, respectively. A user can add custom cognostics or panel functions, if desired. Further details for generating a Trelliscope display with the *ftmsRanalysis* package are provided in the package vignette.

## Results

We demonstrate many of the primary data processing, filtering and visualization functionality enabled by *ftmsRanalysis* on a FT-MS dataset from a soil microbiology study. The experiment consisted of 10 samples taken from two locations: Wisconsin and Michigan. Within each of these locations, half of the samples came from plots of continuous corn cultivation and the other half from plots of switchgrass cultivation. Detailed information about sampling locations and soil characteristics were described previously [29]. A 12 Tesla Bruker SolariX FT mass spectrometer was used to collect high-mass resolution and mass accuracy spectra of the organic molecules in the soil extracts. More detailed information about extraction procedures, instrumentation, and molecular formula assignment can be found in [6]; peaks were aligned between samples using m/z and the default Formularity [17] tolerance parameter. The masses, peak intensities and formulae assignments are available in an Excel file as S1 File, and the R code used in subsequent data analyses are available as S2 File.

**Fig 1:**
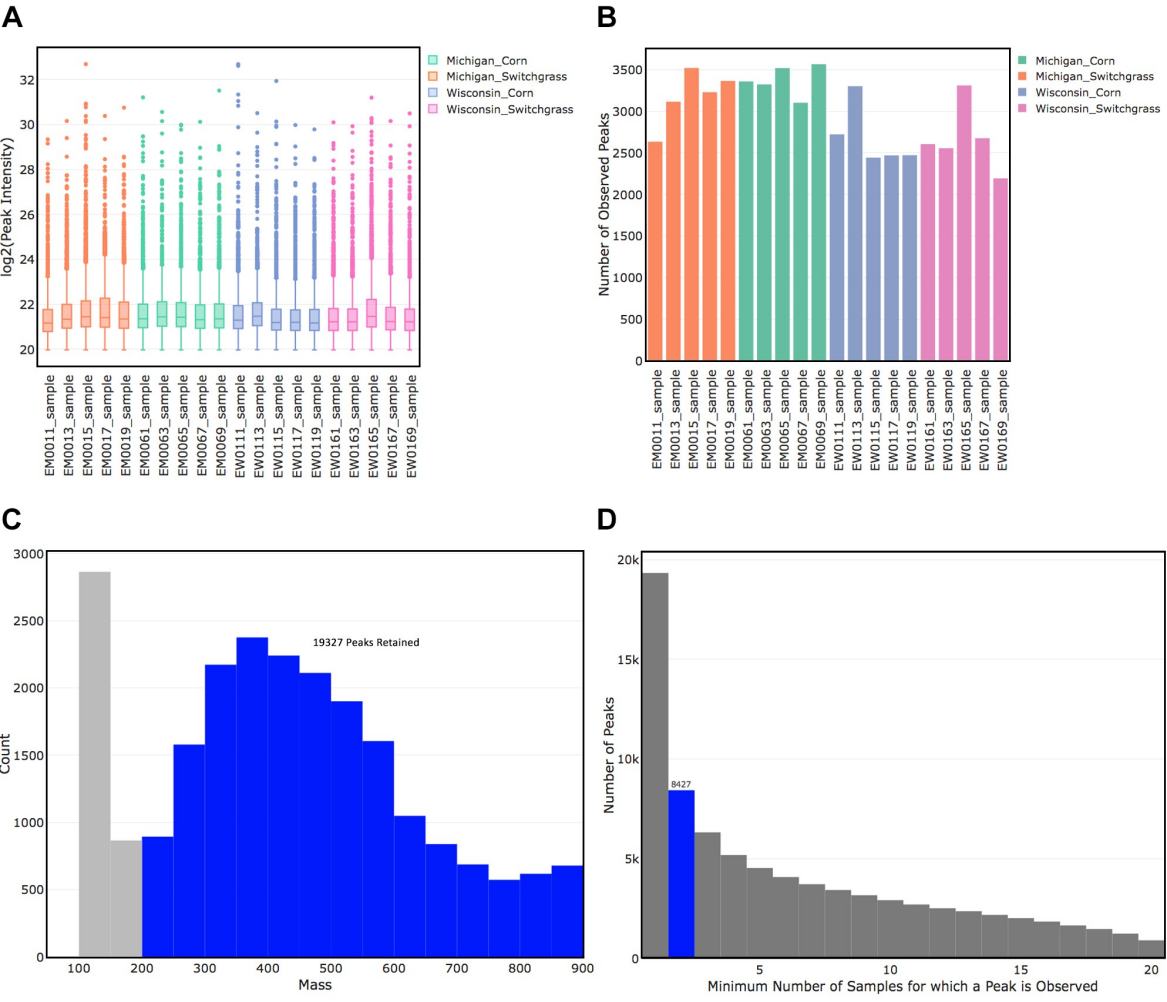
(A) Log2-transformed peak intensity profiles for each sample colored by group. (B) Number of peaks observed in each sample colored by group. (C) Histogram of all observed masses, with masses colored in blue representing those that would be retained after application of a mass_filter(). (D) Barchart corresponding to the minimum number of samples for which a peak was observed, with the height of the bar giving the number of peaks for which this is true for 1 to 20 samples. The highlighted bar gives the number of peaks remaining if the molecule_filter() requiring a peak to be observed in two or more samples is applied.

Data were imported into R and used to create a peakIcrData object. Experimental groups of interest were then specified by indicating the location and crop/flora type variables as the main effects of interest, resulting in 5 samples per group. Fig 1A and 1B give the plots created by the plot() function on the log_2_-transformed peak intensities and the presence/absence transformed data. The initial FT-MS data set contained 20 samples with 23,060 peaks observed in at least one of the samples and 81.74% of peaks absent among the samples; of the 23,060 peaks, 50.59% had formulae assigned. A mass filter was applied to retain peaks with an observed mass between 200 and 900, the trusted mass range based on instrument calibration data. The resulting data had 19,327 peaks and 79.30% of peaks absent among the samples. Next, a filter was applied to the data to retain peaks observed in at least two samples, resulting in data with 8,427 peaks and 59.99% of peaks absent among the samples. Fig 1C and 1D show the effect of implementing the mass and molecule filters, respectively. Finally, a filter was applied to retain only those peaks for which a molecular formula could be assigned. The final dataset contained 6,498 peaks observed over 20 samples and 54.24% of peaks absent among the samples.

**Fig 2:**
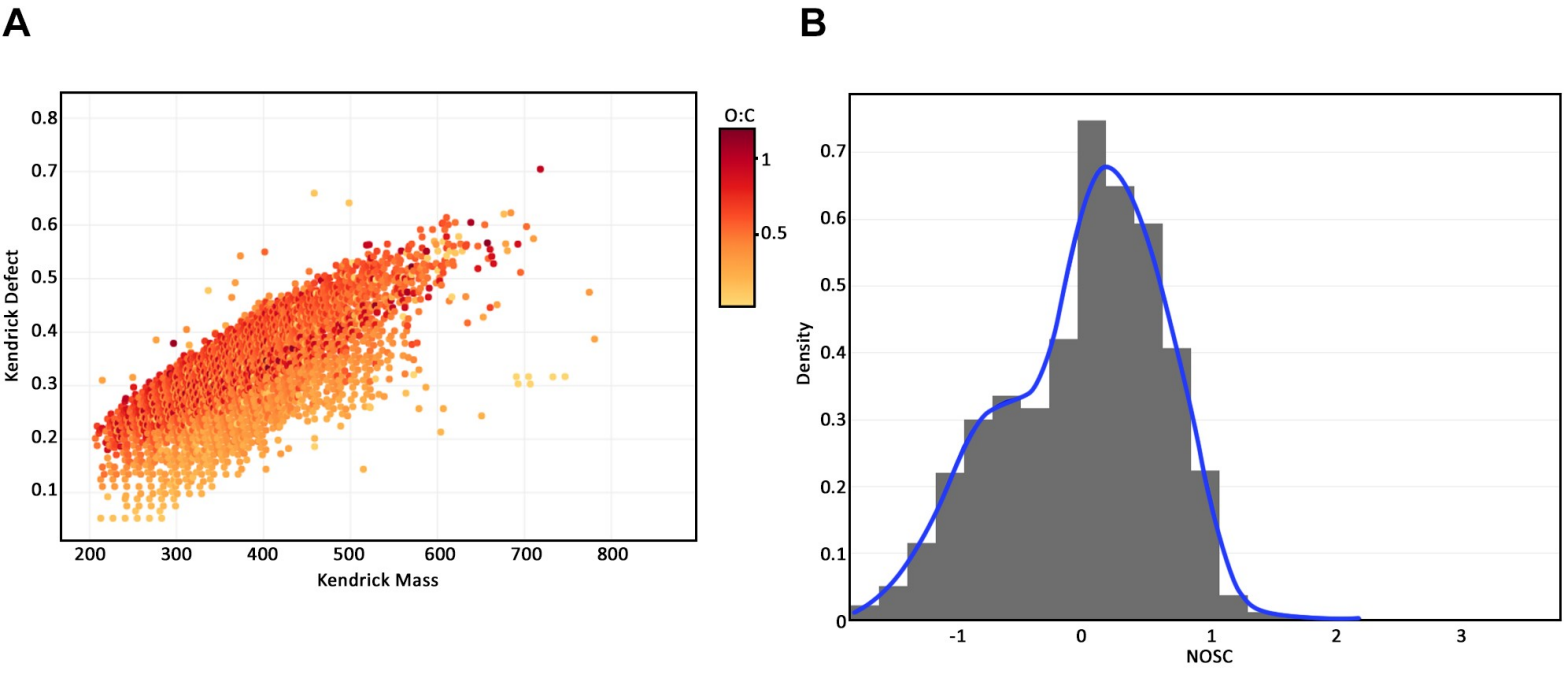
(A) Kendrick plot for EM0011_sample. (B) NOSC histogram and density curve for observed peaks for the EM0011_sample.

**Fig 3:**
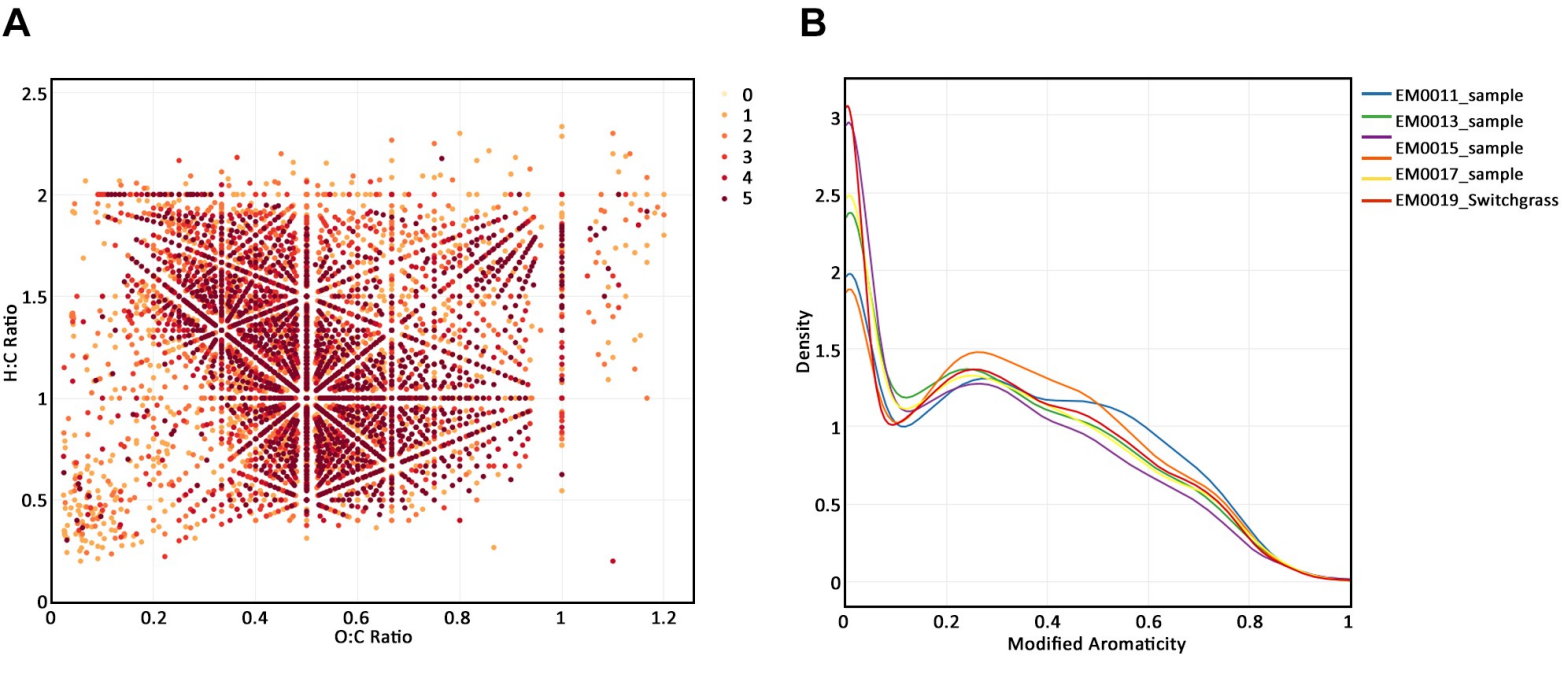
(A) Van Krevelen plot for Michigan switchgrass samples with points colored by the number of samples in which the compound was observed colored by number of observations. (B) Individual sample and group-level modified AI density curves for Michigan switchgrass.

**Fig 4:**
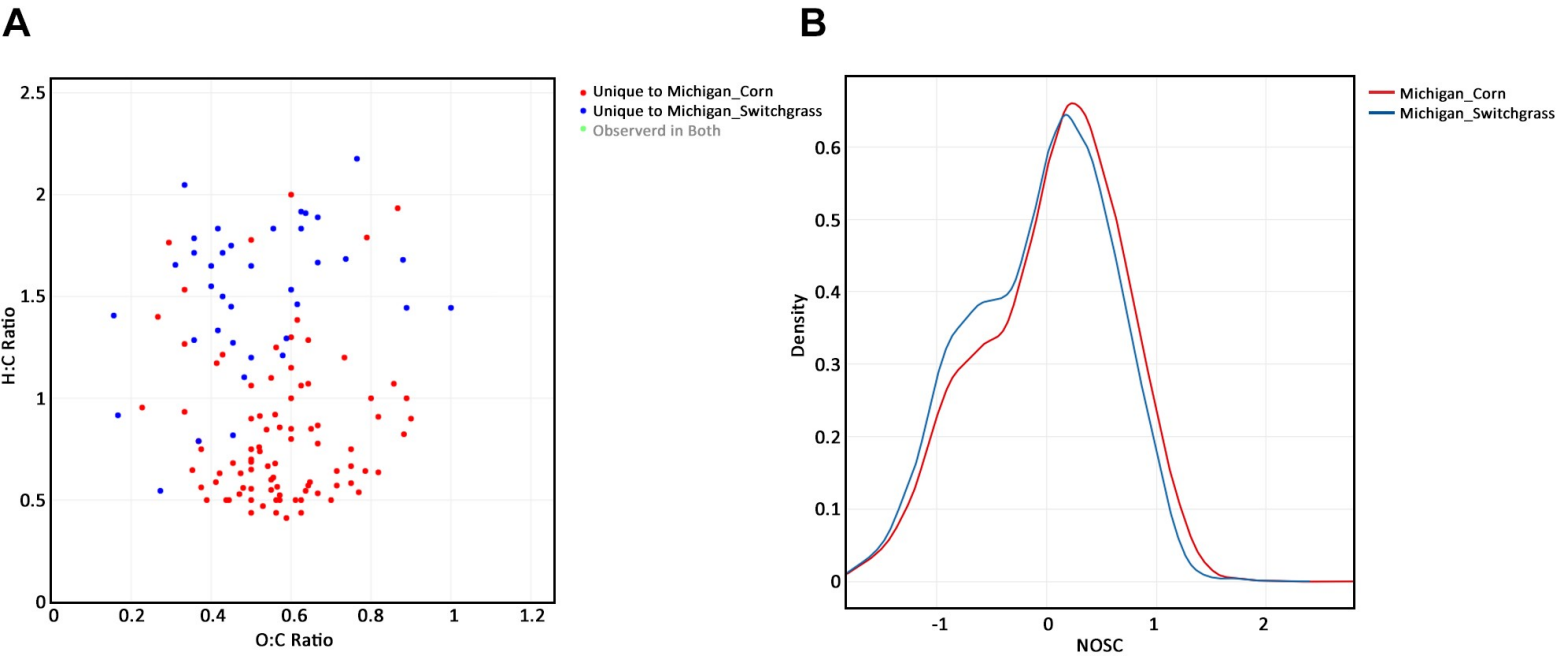
(A) Van Krevelen plot of peaks uniquely observed in Michigan corn and Michigan switchgrass samples. (B) NOSC density curves for Michigan corn and switchgrass groups.

After filtering, the elemental composition and ratios, Kendrick mass and defect, AI, modified AI and NOSC were calculated for each mass with an assigned formula. Plots using data from a single sample, multiple samples belonging to the same group, and a comparison of samples from two groups were created using a few lines of code with *ftmsRanalysis*. Fig 2A and 2B show a Kendrick plot and a density plot of the observed NOSC values for a single sample from the switchgrass plot in Michigan, respectively. Fig 3A shows a van Krevelen plot of the Michigan switchgrass samples; masses not observed in this subset of samples were deselected from the plotly graph. Fig 3B shows the density curves of modified AI values for these samples as well as the group-level density curve. A comparison of Michigan switchgrass and corn samples was conducted via a qualitative G-test to determine which masses present were unique to each crop type in the Michigan samples (using a p-value threshold of 0.05), requiring that an identified mass was observed in more than half the samples to be considered present in a group. Fig 4A shows a van Krevelen plot of the compound formulae found to be unique to each crop type, with the compounds observed in both groups deselected in plotly. Compounds unique to Michigan switchgrass tended to have a higher H:C ratio than those uniquely observed in Michigan corn. Fig 4B shows the densities of the group-level NOSC where compounds in the Michigan corn samples tended to have higher observed NOSC values than those in the Michigan switchgrass samples.

**Fig 5:**
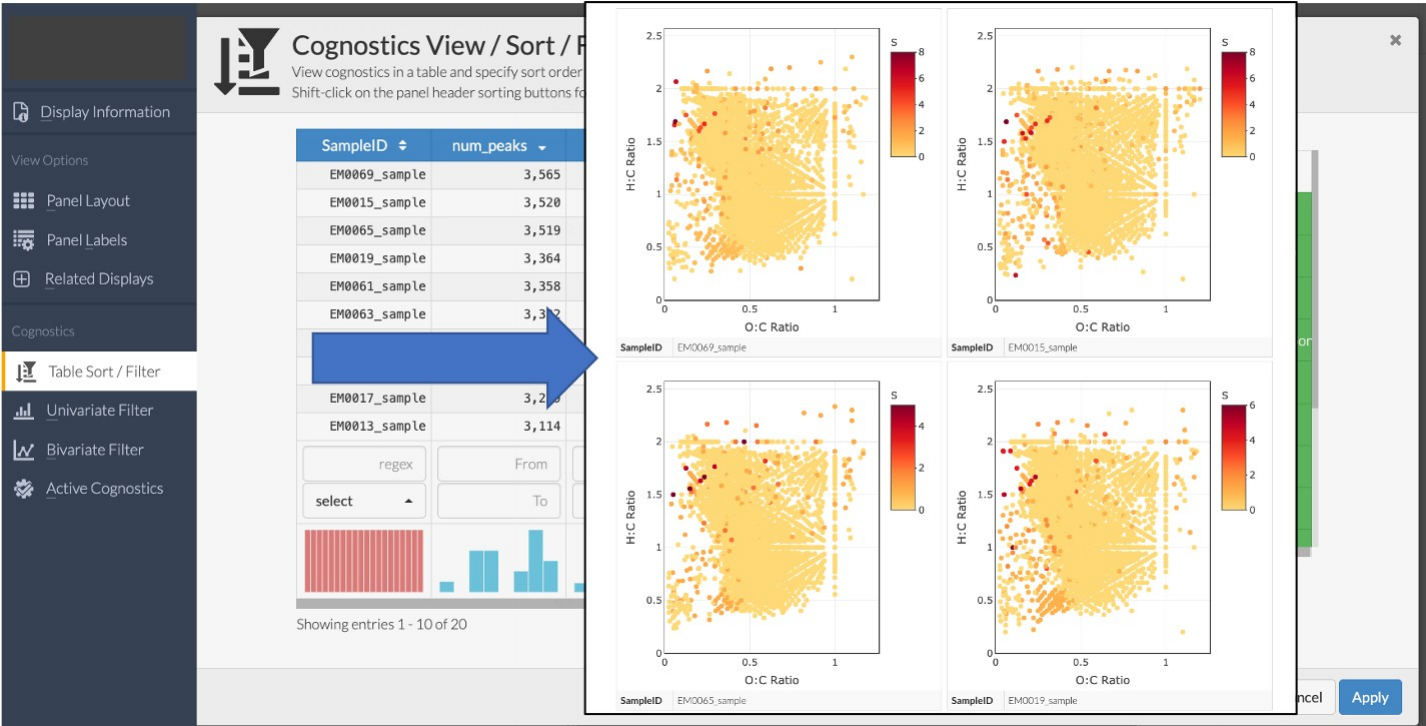
Screenshot of a Trelliscope display showing a van Krevelen plot for each sample. The left image shows the sort/filter capability, with which characteristics of each sample (e.g. number of observed formulae) can be used to order plots. The right image shows the first page of 2x2 plots of individual samples ordered by number of observed compounds and colored by number of S atoms.

Experiments that involve many FT-MS runs generate large datasets that can potentially yield hundreds of plots during an analysis. To facilitate interactive visual exploration of the data, we generated overviews of all samples in the dataset using Trelliscope [28], rather than generating tens to hundreds of static images for each sample and the groups of samples. The capability of *ftmsRanalysis* to easily create Trelliscope displays is shown by dividing the data by sample, specifying a van Krevelen plot display function, and specifying the default cognostics function for van Krevelen displays. Fig 5 shows two screenshots of the resulting Trelliscope user interface for these data and specifications, highlighting the ability to sort and display plots based on user specifications.

## Availability and future directions

The *ftmsRanalysis* open-source software package is implemented in R and available for download via Github (http://github.com/EMSL-Computing/ftmsRanalysis). Additionally, installation instructions, tutorials, and detailed vignettes are available at https://emsl-computing.github.io/ftmsRanalysis/. The *MetaCycData* R package, used with *ftmsRanalysis*, for metabolic mapping is available via Github (https://github.com/EMSL-Computing/MetaCycData). The *ftmsRanalysis* package is a collection of R functions that enable data formatting and preprocessing, filtering, calculation of chemical properties, exploratory data analysis and visualization of FT-MS data. There are several natural extensions of the *ftmsRanalysis* package: implementing the package capabilities in a user interface (e.g. a Shiny application) for domain scientists and adding further statistical tests and methods, particularly for examining data in a quantitative manner. For specific experimental or data applications, users may also wish to create or contribute custom plots and metrics to the code repository.

## Supporting information

S1 File Soil microbiology dataset files for *ftmsRanalysis*. (XLSX)

S2 File R code for data analysis. (R)
